# Design and Demonstration of Impedance-matched Dual-band Chiral Metasurfaces

**DOI:** 10.1038/s41598-018-20056-2

**Published:** 2018-02-22

**Authors:** Minseok Kim, George V. Eleftheriades

**Affiliations:** The Edward S. Rogers Sr. Department of Electrical and Computer Engineering, University of Toronto, Toronto, ON M5S 3G4 Canada

## Abstract

We propose a new family of impedance-matched chiral metasurfaces that offer arbitrary polarization control at two different frequencies. To this end, two main problems are addressed: (1) determination of the required surface impedances for a certain user-defined chiral functionality at two frequencies and (2) their physical realization at microwaves. The first milestone is achieved through a proposed synthesis method that combines a semi-analytical method and a nonlinear optimization technique. In particular, the impedances are computed such that the devised chiral metasurface is also impedance-matched to a terminating medium. The chiral metasurfaces are then physically realized at microwaves by cascading layers of rotated arrays of multiple concentric rectangular copper rings. We establish that these proposed unit cells offer distinct dual-resonances that can be arbitrarily and independently tuned for two orthogonal linear polarizations at each of the two operating frequencies. This allows simultaneous physical mapping of the required surface impedances at two frequencies. The versatility and generality of the proposed numerical and physical solutions are demonstrated through two design examples: A dual-band circular polarization selective surface (CPSS) and a dual-band polarization rotator (PR). The dual-band CPSS is further confirmed experimentally at 20 GHz and 30 GHz based on a free-space quasi-optical system.

## Introduction

The idea of using artificial surfaces, or metasurfaces to control various aspects of scattered electromagnetic (EM) waves has gained much interest due to their exotic beam manipulation capabilities within a low profile. These surfaces consist of arrays of subwavelength-sized scatterers, or unit cells that locally interact with electric, magnetic, or both components of an incident field to manipulate the state of the scattered EM waves^1–8^. Various metasurfaces have been demonstrated in which the main goal has been to manipulate the wavefront of scattered EM waves such that an incident beam is custom reflected/refracted^2–4,6,8^, focused^5^, or re-shaped^7^. The unit cells in these surfaces are adjusted to encode certain phase/amplitude profiles across the surface to engineer the scattered wavefront. While these phase/amplitude metasurfaces have been extensively investigated, another important aspect to consider is the ability to arbitrarily control the polarization state of the scattered waves. This is of particular importance in many applications both in the microwave and optical regimes. In this regard, microwave and optical birefringent metasurfaces have been proposed^7,9–11^. These birefringent metasurfaces are similar to the phase/amplitude metasurfaces except that they utilize anisotropic unit cells which scatter an incident field with two different reflection/transmission phases for two orthogonal linear polarizations. Despite their successful demonstration as quarter or half waveplates, they do not offer complete polarization control of EM waves. This is because they fail to control the flow (e.g., phase velocity) of different circular polarizations (CPs). Such a functionality, however, can be realized with chirality, which allows controlling the flow of both left-handed circular polarization (LHCP) and right-handed circular polarization (RHCP). Therefore, it is possible to realize a circular polarization selective surface (CPSS) with such chiral metasurfaces, which transmit one handedness of CP, while reflecting the opposite handedness. Such a CPSS is of particular interest in satellite communications as it would allow reducing the number of main reflectors required for generating multiple beam patterns^12^. Another useful chiral metasurface application includes a polarization rotator (PR), which allows rotating an incoming linearly polarized wave by any angle of choice regardless of the incident polarization plane.

Motivated from such applications that require chirality, chiral metasurfaces or metasurfaces that mimic chirality have been previously demonstrated both in the microwave and optical regimes. It should be noted that we use the term ‘chiral metasurfaces’ for those devices that control the flow of a RHCP and a LHCP field in the sense that the eigenmodes of a chiral medium are circularly polarized. For example, a linear polarizer sandwiched between two circular polarizers has been demonstrated to function as a CPSS^13^ in the microwave regime. Nonetheless, these require many layers and only operate in a single frequency band. On the other hand, a CPSS^14^ and a PR^15^ have been demonstrated in the optical regime by cascading identical unit cells that are progressively rotated along the propagating direction of an incident field. The operation of these so-called ‘twisted-metamaterials’ is akin to mimicking a helical structure which is a known geometry for chiral molecules found in nature^16^. However, because they rely on a rotated lattice effect such that the chirality becomes realizable in a simple way, the corresponding structures are not necessarily optimal, in the sense that they are not always impedance-matched, which results to degraded efficiency from undesired reflections^14,15^. Likewise, microwave chiral metasurfaces comprising arrays of Pierrot unit cells^12^ and multiple layers of progressively rotated meander lines^17^ have been proposed. While the meander lines are progressively rotated, their dimensions from layer to layer are not identical and they are optimized to satisfy the impedance-matching condition. However, they are single-banded while many antenna applications require metasurfaces that can operate at two different frequency bands^18,19^. For example, certain communication satellites have two operating frequency bands for uplink and downlink that are centered around 20 GHz and 30 GHz. In these applications, CP is preferred, because alignment between transmitting and receiving antennas is then not needed. Therefore, it naturally draws interest in developing a dual-band chiral metasurface configured as a dual-band CPSS. Very recently, multiple layers of progressively rotated meander lines have been further investigated numerically for such a purpose^19^. Nevertheless, their dual-band chiral functionality is fixed to CP selectivity. In other words, it is not feasible to realize a dual-band chiral functionality other than dual-band CP selectivity (e.g., a dual-band PR). It should further be mentioned that their CP selectivity is also not general in the sense that the handedness of the reflected CP waves is fixed. Specifically, if a LHCP field is reflected at one of the operating frequencies, then a RHCP field is reflected at the other frequency and vice versa. Therefore, such a method does not allow a dual-band CPSS that only reflects a LHCP field at its two operating frequencies. Furthermore, they require many layers and their two operating frequencies also depend on the number of layers used. On the other hand, Selvanayagam *et al*., Kim *et al*., and Pfeiffer *et al*. have recently demonstrated general impedance-matched chiral metasurfaces based on multiple layers of tensor impedance surfaces in the microwave and optical regimes^20–23^. While their chiral functionality and operating frequency can be user-defined, they are single-banded which limits their applications.

In departure from the aforementioned works, this paper proposes general dual-band chiral metasurfaces that are also impedance-matched. Preliminary numerical results for an impedance-matched dual-band CPSS have been reported in^24^. Here, we further demonstrate the versatility and generality of our approach by presenting the detailed design of two examples: A dual-band CPSS and a dual-band PR that are impedance-matched and operate at 20 GHz and 30 GHz. Experimental results for the impedance-matched dual-band CPSS are also presented here. These devices are based on only four cascaded layers of tensor impedance surfaces. Each of these layers consists of arrays of multiple concentric rectangular metallic (copper) rings and each layer is rotated with respect to one another. However, they are not progressively rotated and only a few layers are used, unlike the aforementioned previous works^13–15,19^. Instead, we shall show that the hereby proposed unit cell geometries and their interlayer rotation angles are precisely determined such that they encode specific surface impedance values for a certain user-defined dual-band chiral functionality with maximum efficiency. By maximum efficiency, we specifically mean that the devised dual-band chiral metasurface is also impedance matched such that unwanted reflections are minimized.

## Results

### Synthesis of impedance-matched dual-band chiral metasurfaces: Dual-band CPSS and dual-band PR

**Figure 1:**
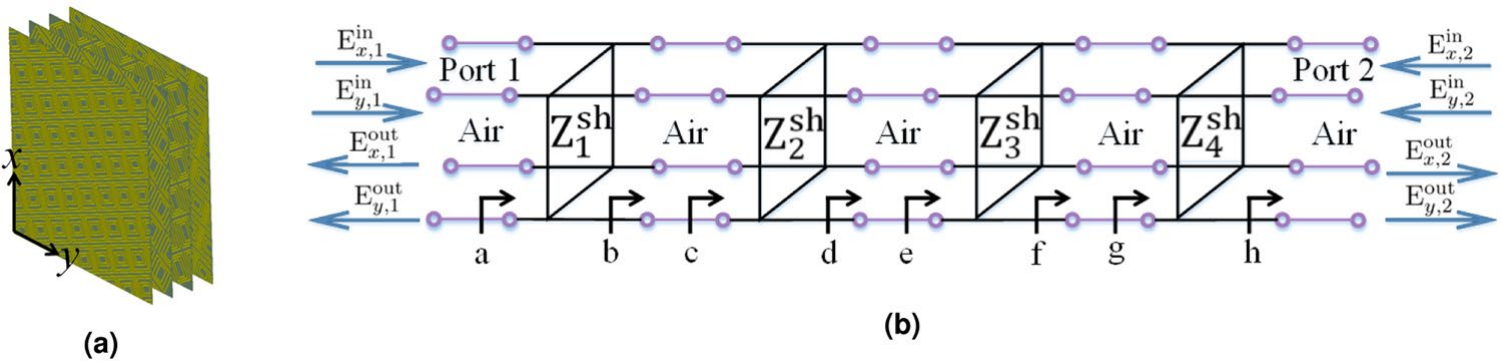
(**a**) The layout of cascaded layers of tensor impedance surfaces comprising a dual-band chiral metasurface and (**b**) its equivalent multi-conductor transmission-line circuit model.

To demonstrate a general dual-band chiral metasurface that is also impedance-matched, we first begin by discussing its synthesis method based on a design example of a dual-band CPSS operating at *f*_1_ = 20 GHz and *f*_2_ = 30 GHz. As we shall show, the synthesis method can be applied to other dual-band chiral functionalities and combines both semi-analytical and nonlinear optimization methods. For an impedance-matched dual-band CPSS, we utilize four tensor impedance surfaces that are separated by 3 mm as shown in Fig. 1a. Different number of layers can be considered. However, one cannot employ less than three layers for realizing a CPSS as theoretically explained in^20^. In other words, even for a single band of operation, at least three cascaded layers are required^20^. Here, an additional layer is inserted to increase the degrees of freedom. The goal now is to determine the required impedances in each layer at *f*_1_ and *f*_2_ such that the cascaded layers operate as an impedance-matched CPSS at the two frequencies. There are two main steps for achieving this goal: (1) determination of the surface impedances at *f*_1_ and the rotation angle of each layer that make the cascaded layers an impedance-matched CPSS at *f*_1_ and (2) determination of the surface impedances at *f*_2_ that make the cascaded layers an impedance-matched CPSS at *f*_2_ with the same rotation angles found in the previous step.

Following these two main steps, we first analyze the four-layered system at *f*_1_. To this end, we refer to the analysis outlined in^20^ from where the key steps are summarized here. Specifically, an equivalent circuit model based on the multi-conductor transmission line (MTL) theory is used to describe the system as shown in Fig. 1b. Here, each tensor impedance layer is modeled as a shunt network, while the air gaps between the layers are modeled as four-wired transmission-lines having the intrinsic characteristic impedance. The circuit physically consists of one input and one output port, yet two orthogonal linear polarizations (linearly-polarized fields along the *x*- and *y*-directions) at each port are treated separately. Hence, the whole circuit is regarded as a four-port system. As such, the input and output relationship is expressed in terms of the scattering matrices (S-matrices) in a linear polarization basis as,

1 $$[\begin{array}{c}{{\rm{E}}}_{x\mathrm{,1}}^{{\rm{out}}}\\ {{\rm{E}}}_{y\mathrm{,1}}^{{\rm{out}}}\\ {{\rm{E}}}_{x\mathrm{,2}}^{{\rm{out}}}\\ {{\rm{E}}}_{y\mathrm{,2}}^{{\rm{out}}}\end{array}]={\bf{S}}[\begin{array}{c}{{\rm{E}}}_{x\mathrm{,1}}^{{\rm{in}}}\\ {{\rm{E}}}_{y\mathrm{,1}}^{{\rm{in}}}\\ {{\rm{E}}}_{x\mathrm{,2}}^{{\rm{in}}}\\ {{\rm{E}}}_{y\mathrm{,2}}^{{\rm{in}}}\end{array}]$$

where the superscripts “in” and “out” respectively denote whether a field is an input or an output. The subscripts *x* and *y* respectively represent the polarization direction of the fields. The subscript number (1 and 2) denotes the port number. The 4 × 4 S-matrix, **S**, describes the equivalent MTL circuit and it is in the form given by,

2 $$\begin{array}{l}{\bf{S}}=[\begin{array}{cc}{{\boldsymbol{\Gamma }}}_{{\rm{A}}} & {{\bf{T}}}_{{\rm{B}}}\\ {{\bf{T}}}_{{\rm{C}}} & {{\boldsymbol{\Gamma }}}_{{\rm{D}}}\end{array}]\end{array}$$

where

3 $$\begin{array}{lllll}{{\boldsymbol{\Gamma }}}_{{\rm{A}}} & = & [\begin{array}{cc}{\rm{S}}\mathrm{(1111)} & {\rm{S}}\mathrm{(1112)}\\ {\rm{S}}\mathrm{(1211)} & {\rm{S}}\mathrm{(1212)}\end{array}]\quad {{\bf{T}}}_{{\rm{B}}} & = & [\begin{array}{cc}{\rm{S}}\mathrm{(1121)} & {\rm{S}}\mathrm{(1122)}\\ {\rm{S}}\mathrm{(1221)} & {\rm{S}}\mathrm{(1222)}\end{array}]\\ {{\bf{T}}}_{{\rm{C}}} & = & [\begin{array}{cc}{\rm{S}}\mathrm{(2111)} & {\rm{S}}\mathrm{(2112)}\\ {\rm{S}}\mathrm{(2211)} & {\rm{S}}\mathrm{(2212)}\end{array}]\quad {{\boldsymbol{\Gamma }}}_{{\rm{D}}} & = & [\begin{array}{cc}{\rm{S}}\mathrm{(2121)} & {\rm{S}}\mathrm{(2122)}\\ {\rm{S}}\mathrm{(2221)} & {\rm{S}}\mathrm{(2222)}\end{array}]\end{array}$$

The scattering parameters are defined as S (outputport *m*, output mode *i*, input port n, input mode *j*) where *m*, *n*, *i*, and *j* are either 1 or 2. We note that mode 1 and mode 2 refer to the fields that are polarized along the *x*- and *y*-directions respectively. For example, S (2111) indicates S(outputport 2, outputmode 1, inputport 1, inputmode1) and defines the field ratio between mode 1 at port 2 to mode 1 at port 1, assuming that it is the only input. On the other hand, more compact 2 × 2 impedance and admittance matrices (**Z** and **Y**) can be defined to describe each tensor impedance layer, which is in a form given by,

4 $$\begin{array}{rcl}{\bf{Z}} & = & [\begin{array}{cc}{{\rm{Z}}}_{11}^{{\rm{sh}}} & {{\rm{Z}}}_{12}^{{\rm{sh}}}\\ {{\rm{Z}}}_{21}^{{\rm{sh}}} & {{\rm{Z}}}_{22}^{{\rm{sh}}}\end{array}]\\  & = & {\bf{R}}({\theta }_{n}){\bf{Z}}({\theta }_{n}=\mathrm{0)}{{\bf{R}}}^{-1}({\theta }_{n})\\  & = & {{\bf{Y}}}^{-1},\end{array}$$

where ${{\rm{Z}}}_{ij}^{{\rm{sh}}}$ represents the shunt impedance of the *n*^th^ layer in which *i* and *j* are again either 1 or 2 for representing mode 1 or 2. **R** is a rotational matrix and *θ*_*n*_ is the angle for which the *n*^th^ layer is rotated by. In other words, **R** is a square matrix whose columns are the linearly independent eigenvectors of one of **Z** and **Y**. Moreover, the form given in (4) can also be used to define the input impedance and admittance anywhere along the equivalent circuit shown in Fig. 1b for which it is related to **Γ**_A_ as

5a $${\bf{Z}}={{\rm{Z}}}_{{\rm{o}}}({\bf{I}}+{{\boldsymbol{\Gamma }}}_{{\rm{A}}})\cdot {({\bf{I}}-{{\boldsymbol{\Gamma }}}_{{\rm{A}}})}^{-1}$$

5b $${{\boldsymbol{\Gamma }}}_{{\rm{A}}}=({\bf{Z}}-{{\bf{Z}}}_{{\rm{ref}}})\cdot {({\bf{Z}}+{{\bf{Z}}}_{{\rm{ref}}})}^{-1}$$

5c $$\,\,\,\,\,\,\,=\,({{\bf{Y}}}_{{\rm{ref}}}-{\bf{Y}})\cdot {({{\bf{Y}}}_{{\rm{ref}}}+{\bf{Y}})}^{-1},$$

where **Z**_ref_ is a diagonal matrix having the intrinsic characteristic impedance of Z_o_ as its non-zero component and ${{\bf{Y}}}_{{\rm{ref}}}={{\bf{Z}}}_{{\rm{ref}}}^{-1}$.

In this first part of the analysis at *f*_1_, it is first assumed that the surface impedances for the first and last layers are known. The rest of the unknown impedance values for the middle two layers are solved for such that the net S-matrix of the cascaded layers matches to that of an ideal CPSS (**S**_CPSS_) which is given by,

6 $${{\bf{S}}}_{{\rm{CPSS}}}=[\begin{array}{cccc}-1 & j & 1 & -j\\ j & 1 & j & 1\\ 1 & j & -1 & -j\\ -j & 1 & -j & 1\end{array}]0.5{{\rm{e}}}^{j\varphi },$$

where *ϕ* is an arbitrary phase constant. From (6), it is seen that the transmission of a LHCP field is identically 1 (i.e., impedance-matched and lossless), while that of a RHCP field is identically 0 (i.e., perfect reflection). Furthermore, the axial ratio for the transmitted LHCP field and the reflected RHCP field ideally remain unity, which implies that a LHCP field is transmitted into a pure LHCP field, while a RHCP field is reflected as a pure RHCP field.

To determine the unknown impedances of the middle two layers, we first note that (6) represents the desired S-matrix of the overall system. Therefore, the desired co-polarized and cross-polarized input reflection coefficients at point “a” (refer to Fig. 1b) is the first quadrant of (6) (i.e., **Γ**_A_ of **S**_CPSS_). The input admittance at point a, **Y**_a_, can then be solved via (5). Furthermore, because the admittance of the first layer, **Y**_1_, is assumed to be known, the input admittance at point b, **Y**_b_, is given as,

7 $${{\bf{Y}}}_{{\rm{b}}}={{\bf{Y}}}_{{\rm{a}}}-{{\bf{Y}}}_{{\rm{1}}}$$

Similarly, the input admittance at point g, **Y**_g_, can be found because the admittance of the last layer, **Y**_4_, and the input admittance at point h are known (**Y**_h_ = **Y**_ref_). On the other hand, **Y**_b_ and **Y**_g_ are respectively related to the input admittance at points c (**Y**_c_) and f (**Y**_f_) via the phase shift of the air gaps^20^. For example,

8 $${{\bf{Y}}}_{{\rm{f}}}=\frac{1}{{{\rm{Z}}}_{{\rm{o}}}}({{\bf{Y}}}_{{\rm{g}}}+j{{\bf{Y}}}_{{\rm{ref}}}\,\tan (\beta t))({{\bf{Y}}}_{{\rm{ref}}}+j{{\bf{Y}}}_{{\rm{g}}}\,\tan (\beta t{))}^{-1}\mathrm{.}$$

Once **Y**_c_ and **Y**_f_ are obtained, the input admittance at point e, **Y**_e_, is solved via the algebraic Ricatti equation given as^20^,

9 $$\begin{array}{l}-\frac{1}{{{\rm{Z}}}_{{\rm{o}}}}{\rm{Re}}{({{\bf{Y}}}_{{\rm{f}}})}^{-1}\cdot {\rm{Im}}({{\bf{Y}}}_{{\rm{e}}})-\frac{1}{{{\rm{Z}}}_{{\rm{o}}}}{\rm{Im}}({{\bf{Y}}}_{{\rm{e}}})\cdot {\rm{Re}}{({{\bf{Y}}}_{{\rm{f}}})}^{-1}\\ \quad +\,\tan (\beta t){\rm{Im}}({{\bf{Y}}}_{{\rm{e}}})\cdot {\rm{Re}}{({{\bf{Y}}}_{{\rm{f}}})}^{-1}\cdot {\rm{Im}}({{\bf{Y}}}_{{\rm{e}}})+\frac{1}{{{\rm{Z}}}_{{\rm{o}}}^{2}\,\tan (\beta t)}{\rm{Re}}{({{\bf{Y}}}_{{\rm{f}}})}^{-1}\\ \quad +\,\tan (\beta t){\rm{Re}}({{\bf{Y}}}_{{\rm{f}}})-\frac{1}{{{\rm{Z}}}_{{\rm{o}}}}{\rm{Re}}{({{\bf{Y}}}_{{\rm{c}}})}^{-1}\cdot (\frac{1}{{{\rm{Z}}}_{{\rm{o}}}\,\tan (\beta t)}+\frac{\tan (\beta t)}{{{\rm{Z}}}_{{\rm{o}}}}){\bf{I}}={\bf{0}}\end{array}$$

where *t* is the separation length between the tensor impedance layers, which is fixed to 3 mm, and *β* is the free-space wavenumber. Re() and Im() respectively imply the real and imaginary parts of the admittance matrices and **I** is an identity matrix. The Ricatti equation in (9) assumes that each layer is lossless. However, such an assumption is valid in the microwave regime because the complex-valued surface impedances are dominated by their imaginary components. The Ricatti equation has a well-known analytical method to solve with^25^ and allows identifying the unknown impedances of the middle two layers. Specifically, **Y**_3_ can be obtained by subtracting **Y**_f_ from **Y**_e_ and **Y**_2_ can be solved by subtracting **Y**_c_ by **Y**_d_ which is related to **Y**_e_ via an equation similar to (8). However, we note that these impedances are not necessarily guaranteed to be the correct solution, because the impedances of the first and last layers are arbitrarily assumed. To ensure that they are indeed the correct solution, they must be cascaded and compared with the desired final S-matrix (i.e., ***S***_CPSS_). To cascade all tensor impedance layers, the Z-matrix of the n^th^ layer is first expanded to a 4 × 4 Z-matrix, ${{\bf{Z}}}_{{\rm{n}}}^{{\rm{sh}}}$, in a form given by,

10 $${{\bf{Z}}}_{{\rm{n}}}^{{\rm{sh}}}=[\begin{array}{cccc}{{\rm{Z}}}_{11}^{{\rm{sh}}} & {{\rm{Z}}}_{11}^{{\rm{sh}}} & {{\rm{Z}}}_{12}^{{\rm{sh}}} & {{\rm{Z}}}_{12}^{{\rm{sh}}}\\ {{\rm{Z}}}_{11}^{{\rm{sh}}} & {{\rm{Z}}}_{11}^{{\rm{sh}}} & {{\rm{Z}}}_{12}^{{\rm{sh}}} & {{\rm{Z}}}_{12}^{{\rm{sh}}}\\ {{\rm{Z}}}_{21}^{{\rm{sh}}} & {{\rm{Z}}}_{21}^{{\rm{sh}}} & {{\rm{Z}}}_{22}^{{\rm{sh}}} & {{\rm{Z}}}_{22}^{{\rm{sh}}}\\ {{\rm{Z}}}_{21}^{{\rm{sh}}} & {{\rm{Z}}}_{21}^{{\rm{sh}}} & {{\rm{Z}}}_{22}^{{\rm{sh}}} & {{\rm{Z}}}_{22}^{{\rm{sh}}}\end{array}]$$

which is related to its corresponding S-matrix through the conversion given by,

11 $${\bf{S}}={{\bf{G}}}_{{\rm{ref}}}\cdot ({{\bf{Z}}}_{{\rm{n}}}^{{\rm{sh}}}-{{\bf{Z}}}_{{\rm{ref}}})\cdot {({{\bf{Z}}}_{{\rm{n}}}^{{\rm{sh}}}+{{\bf{Z}}}_{{\rm{ref}}})}^{-1}\cdot {{\bf{G}}}_{{\rm{ref}}}^{-1},$$

where **G**_ref_ is a diagonal matrix having $1\sqrt{{{\rm{Z}}}_{{\rm{o}}}}$ as as the diagonal entries. On the other hand, the air gaps are represented as transmission-line S-matrices, ***S***_TL_, given by^26^,

12 $${{\bf{S}}}_{{\rm{TL}}}=[\begin{array}{cccc}0 & 0 & {{\rm{e}}}^{-j\beta t} & 0\\ 0 & 0 & 0 & {{\rm{e}}}^{-j\beta t}\\ {{\rm{e}}}^{-j\beta t} & 0 & 0 & 0\\ 0 & {{\rm{e}}}^{-j\beta t} & 0 & 0\end{array}]\mathrm{.}$$

With the S-matrices in (11) and (12) that describe each module, the net S-matrix of the cascaded system (**S**_net_) can be analytically computed through the generalized scattering matrix method^26^. The **S**_net_ can then be repeatedly compared with **S**_CPSS_ based on different values of surface impedances in each layer. However, the number of combinations in such a case is impractical. To avoid this, we invoke the symmetry condition of the structure. Specifically, the eigenvalues of the first and last layers, and the second and third layers are set to be the same (i.e., the unrotated Z-matrices of the first and last layers and the second and third layers are the same). This allows the required rotation angles in the third and last layers to be immediately determined from the symmetry, because the rotation angle of the third layer (or the last layer) must be the negative angle of the second layer (or the first layer) in order to comply with the symmetry condition. Therefore, the necessary surface impedances for the third and last layers are completely deduced from the first and second layers. As such, **S**_net_ can be repeatedly compared with ***S***_CPSS_ only by varying the eigenvalues of the first and second layers, and their rotation angles. In this regard, although the first part of the analysis at *f*_1_ is semi-analytical, the proposed method significantly reduces the solution space by (a) only considering the solutions with the desired input reflection coefficient (i.e., **Γ**_A_ of **S**_CPSS_)^20^ and (b) utilizing the symmetry condition of the structure. Table 1 summarizes a solution based on the proposed method from which it is seen that the first and last layers are identical to each other except that the first layer is rotated by 20°, while the last layer is rotated by −20°. Similarly, the second and third layers are the same except that the second layer is rotated by 30°, while the third layer is rotated by −30°. These rotation angles are obtained by substituting the impedance parameters in Table 1 to (4). It should also be noted that displacements of the layers in the *xy* plane do not affect ***S***_net_. This is because each layer is modularized and it is characterized by its own scattering matrix which assumes that the layer infinitely extends in the *xy* plane. As such, ***S***_net_ is insensitive to the displacements of layers in the *xy* plane.

**Table 1:** Required shunt impedances of layers #1, #2, #3, and #4 at *f*_1_ = 20 GHz.

Layer #1	Layer #2	Layer #3	Layer #4
${{\rm{Z}}}_{11}^{{\rm{sh}}}$ = −j210 Ω	${{\rm{Z}}}_{11}^{{\rm{sh}}}$ = −j82 Ω	${{\rm{Z}}}_{11}^{{\rm{sh}}}$ = −j82 Ω	${{\rm{Z}}}_{11}^{{\rm{sh}}}$ = −j210 Ω
${{\rm{Z}}}_{12}^{{\rm{sh}}}$ = −j330 Ω	${{\rm{Z}}}_{12}^{{\rm{sh}}}$ = −j215 Ω	${{\rm{Z}}}_{12}^{{\rm{sh}}}$ = j215 Ω	${{\rm{Z}}}_{12}^{{\rm{sh}}}$ = j330 Ω
${{\rm{Z}}}_{22}^{{\rm{sh}}}$ = j580 Ω	${{\rm{Z}}}_{22}^{{\rm{sh}}}$ = −j330 Ω	${{\rm{Z}}}_{22}^{{\rm{sh}}}$ = −j330 Ω	${{\rm{Z}}}_{22}^{{\rm{sh}}}$ = j580 Ω

Once ${{\bf{Z}}}_{{\rm{n}}}^{{\rm{sh}}}$ and *θ*_*n*_ are determined at *f*_1_, the system is now solved at *f*_2_. For this second step, we must use the same rotation angles, *θ*_*n*_, found in the first step to ensure the same physical structure at *f*_2_. To this end, we employ the nonlinear numerical optimization process as outlined in^21,24^. Specifically, we first construct a cost function by utilizing the MTL theory again to model the overall structure. In particular, the cost function is defined as

13 $${\rm{Cost}}=\,{\rm{Max}}(|{{\bf{S}}}_{{\rm{net}}}-{{\bf{S}}}_{{\rm{goal}}}|)$$

where **S**_goal_ is the desired final S-matrix, which in this case is **S**_CPSS_, and Max() returns the maximum value out of 16 parameters. In obtaining **S**_net_, the rotation matrices for all layers are fixed to **R**(*θ*_*n*_) found in the previous step at *f*_1_, while the eigenvalues of **Z** are varied within ±j800 Ω. We note that a greater range of impedances can be considered. However, this will increase the Ohmic loss and reduce the bandwidth of operation. Similarly to the first part of the analysis, the net S-matrix is then constructed by employing the generalized scattering matrix method^26^.

To minimize the cost function, MATLAB’s built-in optimizer, *fmincon*, is employed. The *fmincon* function is based on the gradient descent method that finds a local minimum, rather than the global minimum, of a scalar function of several variables starting with an initial estimate. Although it finds a local minimum, we have determined that the optimizer typically converges in less than 100 iterations with the given range of shunt impedances of ±j800 Ω. Table 2 summarizes the solution found with the proposed nonlinear optimization method at *f*_2_ for which the cost function is evaluated to be 0.05. We note that if the solution at *f*_1_ is substituted to (13), then it results to 0.08, which again is a small value as desired.

**Table 2:** Required shunt impedances of layers #1, #2, #3, and #4 at *f*_2_ = 30 GHz.

Layer #1	Layer #2	Layer #3	Layer #4
${{\rm{Z}}}_{11}^{{\rm{sh}}}$ = −j330 Ω	${{\rm{Z}}}_{11}^{{\rm{sh}}}$ = j190 Ω	${{\rm{Z}}}_{11}^{{\rm{sh}}}$ = j190 Ω	${{\rm{Z}}}_{11}^{{\rm{sh}}}$ = −j330 Ω
${{\rm{Z}}}_{12}^{{\rm{sh}}}$ = −j240 Ω	${{\rm{Z}}}_{12}^{{\rm{sh}}}$ = j350 Ω	${{\rm{Z}}}_{12}^{{\rm{sh}}}$ = −j350 Ω	${{\rm{Z}}}_{12}^{{\rm{sh}}}$ = j240 Ω
${{\rm{Z}}}_{22}^{{\rm{sh}}}$ = j235 Ω	${{\rm{Z}}}_{22}^{{\rm{sh}}}$ = j597 Ω	${{\rm{Z}}}_{22}^{{\rm{sh}}}$ = j597 Ω	${{\rm{Z}}}_{22}^{{\rm{sh}}}$ = j235 Ω

As discussed above, the required impedances at *f*_1_ and *f*_2_ are respectively obtained via the semi-analytical and nonlinear optimization methods. However, it should be noted that it is possible to avoid solving (9) at *f*_1_ and employ only the nonlinear optimization method to simultaneously minimize (13) at *f*_1_ and *f*_2_. However, the solution space in such a case is large and takes much computational effort. On the contrary, what we have proposed here dramatically minimizes the solution space by (a) only considering the solutions with the desired reflection coefficient (**Γ**_A_ of **S**_CPSS_) at *f*_1_, (b) forcing the eigenvalues of the first and last layers and the second and third layers to be the same at *f*_1_, and (c) only varying the eigenvalues of the first and second layers with fixed eigenvectors at *f*_2_ in the nonlinear optimization step. Therefore, both the first and second analyses at *f*_1_ and *f*_2_ effectively minimize the solution space and significantly reduce the computational effort.

To further demonstrate the generality and versatility of the proposed synthesis method, we aim to realize a dual-band PR which rotates a linearly-polarized wave by 90 ° at *f*_1_ and *f*_2_. For it to rotate any linearly-polarized wave regardless of its incidence polarization plane with the highest possible efficiency, it must satisfy the following three conditions:

- Maximize the cross-polarized transmission
- Minimize the co-polarized reflection (i.e., impedance-matching)
- Achieve 180° phase difference between the cross-polarized transmitted fields

It is noted that the first two conditions guarantee the corresponding system to behave as a PR only for the incident fields that are polarized along the principal axes, while the last condition ensures polarization rotation regardless of the incident polarization plane^20^. The three conditions can be compactly represented as the ideal S-matrix for a PR given by,

14 $${{\bf{S}}}_{{\rm{PR}}}=[\begin{array}{cccc}0 & 0 & 0 & 1\\ 0 & 0 & -1 & 0\\ 0 & -1 & 0 & 0\\ 1 & 0 & 0 & 0\end{array}]\mathrm{.}$$

For the demonstration of dual-band PR, we assume six cascaded layers of tensor impedance surfaces. Similar to a CPSS, a PR must consist of at least four layers even for a single-band operation^20^. Here, the number of layers has been chosen to increase the number of degrees of freedom such that the cost function can be better minimized at *f*_2_. However, it is possible to reduce the number of layers at the cost of a degraded performance as long as it is greater than four. To obtain the required surface impedances at *f*_1_ and *f*_2_, we follow the same synthesis method as before, which is summarized below:

1. Define a chiral operation (e.g., polarization rotation and CP selection) and its ideal S-matrix.
2. Assume that the only unknowns are the middle two layers at *f*_1_.
3. Based on the desired S-matrix and the known impedance values, obtain the unknowns by solving (9) at *f*_1_.
4. Repeat steps #2 and #3 with different initial assumptions, until the actual net S-matrix matches to that of a desired S-matrix at *f*_1_.
5. Extract eigenvectors for all tensor impedance layers (i.e., their rotation angles) at *f*_1_.
6. Use the same eigenvectors for all tensor impedance layers at *f*_2_ and utilize the nonlinear optimization method to minimize (13).

Based on the synthesis methodology listed above, the 6 layers are solved to function as a PR at *f*_1_ and *f*_2_ and the results are summarized in Table 3. The values of the cost function at *f*_1_ and *f*_2_ with the impedance values listed in Table 3 result to 0.145 and 0.04 respectively.

**Table 3:** Required shunt impedances of layers #1, #2, #3, #4, #5, and #6 at *f*_1_ = 20 GHz and *f*_2_ = 30 GHz for a dual-band PR.

	At *f*1	At *f*2
Layer #1	${{\rm{Z}}}_{11}^{{\rm{sh}}}$ = −j690 Ω	${{\rm{Z}}}_{11}^{{\rm{sh}}}$ = −j700 Ω
	${{\rm{Z}}}_{12}^{{\rm{sh}}}$ = 0 Ω	${{\rm{Z}}}_{12}^{{\rm{sh}}}$ = 0 Ω
	${{\rm{Z}}}_{22}^{{\rm{sh}}}$ = j680 Ω	${{\rm{Z}}}_{22}^{{\rm{sh}}}$ = j700 Ω
Layer #2	${{\rm{Z}}}_{11}^{{\rm{sh}}}$ = j330 Ω	${{\rm{Z}}}_{11}^{{\rm{sh}}}$ = j250 Ω
	${{\rm{Z}}}_{12}^{{\rm{sh}}}$ = −j115 Ω	${{\rm{Z}}}_{12}^{{\rm{sh}}}$ = −j105 Ω
	${{\rm{Z}}}_{22}^{{\rm{sh}}}$ = −j300 Ω	${{\rm{Z}}}_{22}^{{\rm{sh}}}$ = −j335 Ω
Layer #3	${{\rm{Z}}}_{11}^{{\rm{sh}}}$ = j220 Ω	${{\rm{Z}}}_{11}^{{\rm{sh}}}$ = j535 Ω
	${{\rm{Z}}}_{12}^{{\rm{sh}}}$ = −j230 Ω	${{\rm{Z}}}_{12}^{{\rm{sh}}}$ = −j315 Ω
	${{\rm{Z}}}_{22}^{{\rm{sh}}}$ = −j330 Ω	${{\rm{Z}}}_{22}^{{\rm{sh}}}$ = −j215 Ω
Layer #4	${{\rm{Z}}}_{11}^{{\rm{sh}}}$ = j60 Ω	${{\rm{Z}}}_{11}^{{\rm{sh}}}$ = j190 Ω
	${{\rm{Z}}}_{12}^{{\rm{sh}}}$ = −j600 Ω	${{\rm{Z}}}_{12}^{{\rm{sh}}}$ = −j605 Ω
	${{\rm{Z}}}_{22}^{{\rm{sh}}}$ = −j150 Ω	${{\rm{Z}}}_{22}^{{\rm{sh}}}$ = −j22 Ω
Layer #5	${{\rm{Z}}}_{11}^{{\rm{sh}}}$ = −j30 Ω	${{\rm{Z}}}_{11}^{{\rm{sh}}}$ = −j80 Ω
	${{\rm{Z}}}_{12}^{{\rm{sh}}}$ = −j270 Ω	${{\rm{Z}}}_{12}^{{\rm{sh}}}$ = −j260 Ω
	${{\rm{Z}}}_{22}^{{\rm{sh}}}$ = j70 Ω	${{\rm{Z}}}_{22}^{{\rm{sh}}}$ = j10 Ω
Layer #6	${{\rm{Z}}}_{11}^{{\rm{sh}}}$ = −j190 Ω	${{\rm{Z}}}_{11}^{{\rm{sh}}}$ = −j235 Ω
	${{\rm{Z}}}_{12}^{{\rm{sh}}}$ = −j175 Ω	${{\rm{Z}}}_{12}^{{\rm{sh}}}$ = −j170 Ω
	${{\rm{Z}}}_{22}^{{\rm{sh}}}$ = j230 Ω	${{\rm{Z}}}_{22}^{{\rm{sh}}}$ = j165 Ω

### Physical realization at microwaves

**Figure 2:**
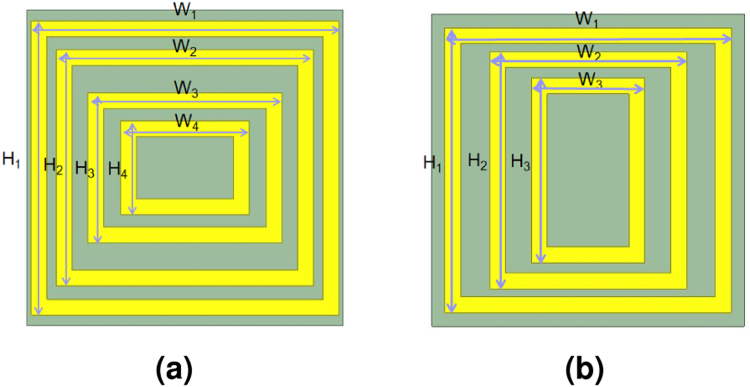
Multiple concentric rectangular rings as a unit cell for a dual-band chiral metasurface. (**a** and **b**) respectively show one period of layer #1 and #4 and layer #2 and #3 for the dual-band CPSS. The copper metalization with the thickness of 18 *μm* is done on a 0.127 mm thick Rogers 5880 substrate and the width of all rectangular rings are fixed to 0.2 mm. The square lattice constant of 4 mm is assumed for all layers. The geometries for layers #1 and #4 are: W_1_ = 3.898 mm, H_1_ = 3.726 mm, W_2_ = 3.256 mm, H_2_ = 2.995 mm, W_3_ = 2.463 mm, H_3_ = 1.903 mm, W_4_ = 1.628 mm, and H_4_ = 1.191 mm. For the layers #2 and #3: W_1_ = 3.667 mm, H_1_ = 3.642 mm, W_2_ = 2.521 mm, H_2_ = 3.03 mm, and W_3_ = 1.448 mm, H_3_ = 2.359 mm.

**Figure 3:**
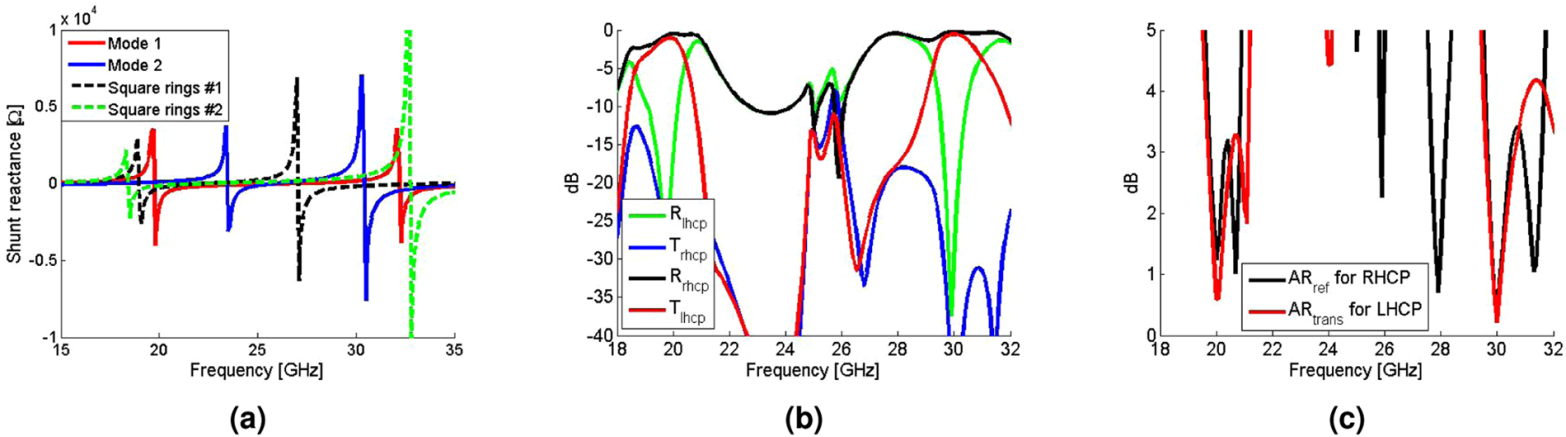
(**a**) An example of the variation of the shunt reactance of the proposed unit cell. The solid and dotted curves respectively represent rectangular and square rings. (**b**) The transmission and reflection of CP waves and (**c**) the axial ratio of the transmitted and reflected CP waves for the dual-band CPSS. R and T respectively represent the reflection and transmission coefficients, and the subscript indicates the handedness. AR_ref_ and AR_trans_ refer to the axial ratio of the reflected and transmitted waves respectively.

With the required surface impedances determined at *f*_1_ and *f*_2_ via the proposed synthesis method, a unit cell is designed to physically encode the required impedances. The required eigenvalues of each Z-matrix for the dual-band CPSS and dual-band PR are either capacitive or inductive. Therefore, to physically encode these impedances, a unit cell must (a) possess two resonances for accessing both capacitive and inductive values near *f*_1_ and *f*_2_ and (b) the two resonant frequencies should be arbitrarily and independently tunable for the two orthogonal linearly-polarized waves (mode 1 and mode 2). To realize such a unit cell, we propose multiple concentric rectangular copper rings as shown in Fig. 2. It has been previously reported that an array of double concentric rectangular rings possesses dual-resonances^27^. However, with only two concentric rings, we find that the unit cell periodicity needs to be large to resonate which results in higher-order Floquet modes. This is not desirable, since our analysis assumes only the fundamental mode propagating. Here, however, we show that having more rectangular rings allows dual-resonances with electrically small unit cell sizes. Specifically, the proposed unit cell periodicity is 4 mm (2.5 times smaller than the smallest operating wavelength) and Fig. 3a shows the variation in surface reactance for different physical geometries. The dotted curves show the variation in case of concentric square rings in which a field that is polarized along the *x*-direction (mode 1) and another field that is polarized along the *y*-direction (mode 2) experience the same physical structure. From Fig. 3a, it can be noted that there exists clear dual-resonances for which their resonant frequencies can be tuned. This specific example demonstrates tuning of the two resonant frequencies from 19 GHz and 27 GHz to 18 GHz and 33 GHz. On the other hand, the blue and red curves respectively show the variation for modes 1 and 2 in case of concentric rectangular rings from which it is seen that the resonant frequencies for the two modes can also be independently tuned. Therefore, the proposed unit cell can simultaneously implement any impedance value at 20 GHz and 30 GHz and the required impedances shown in Tables 1 and 2 can be physically mapped. The details of their geometrical values are found in the caption of Fig. 2.

To evaluate its performance as a dual-band CPSS, the net S-matrix of the cascaded layers with the proposed unit cells is numerically computed. To this end, it is desirable to simulate the whole structure, however this is not feasible because each layer is rotated with respect to one another and the corresponding global periodicity is too large to simulate. Instead, we have simulated a single layer at a time with ANSYS High Frequency Electromagnetic Field Simulation (HFSS) (see Methods) and analytically cascaded all layers through the generalized scattering matrix method^26^. Such an approach does not capture the coupling between the layers. Nonetheless, provided that higher order Floquet modes are sufficiently small, the effect of coupling can be minimized. The result is shown in Fig. 3b in which it is seen that the transmitted LHCP and the reflected RHCP are maximized near 20 GHz and 30 GHz. The transmission coefficients of a LHCP wave at 20 GHz and 30 GHz are respectively −1.1 dB and −0.56 dB, whereas the reflection coefficients for a RHCP wave at 20 GHz and 30 GHz are −0.52 dB and −0.22 dB respectively. Furthermore, the axial ratio shown in Fig. 3c is well below 5 dB near 20 GHz and 30 GHz which confirms that what gets transmitted and reflected remains circularly polarized. Hence, the stack of the proposed unit cells indeed functions as an impedance-matched dual-band CPSS.

**Figure 4:**
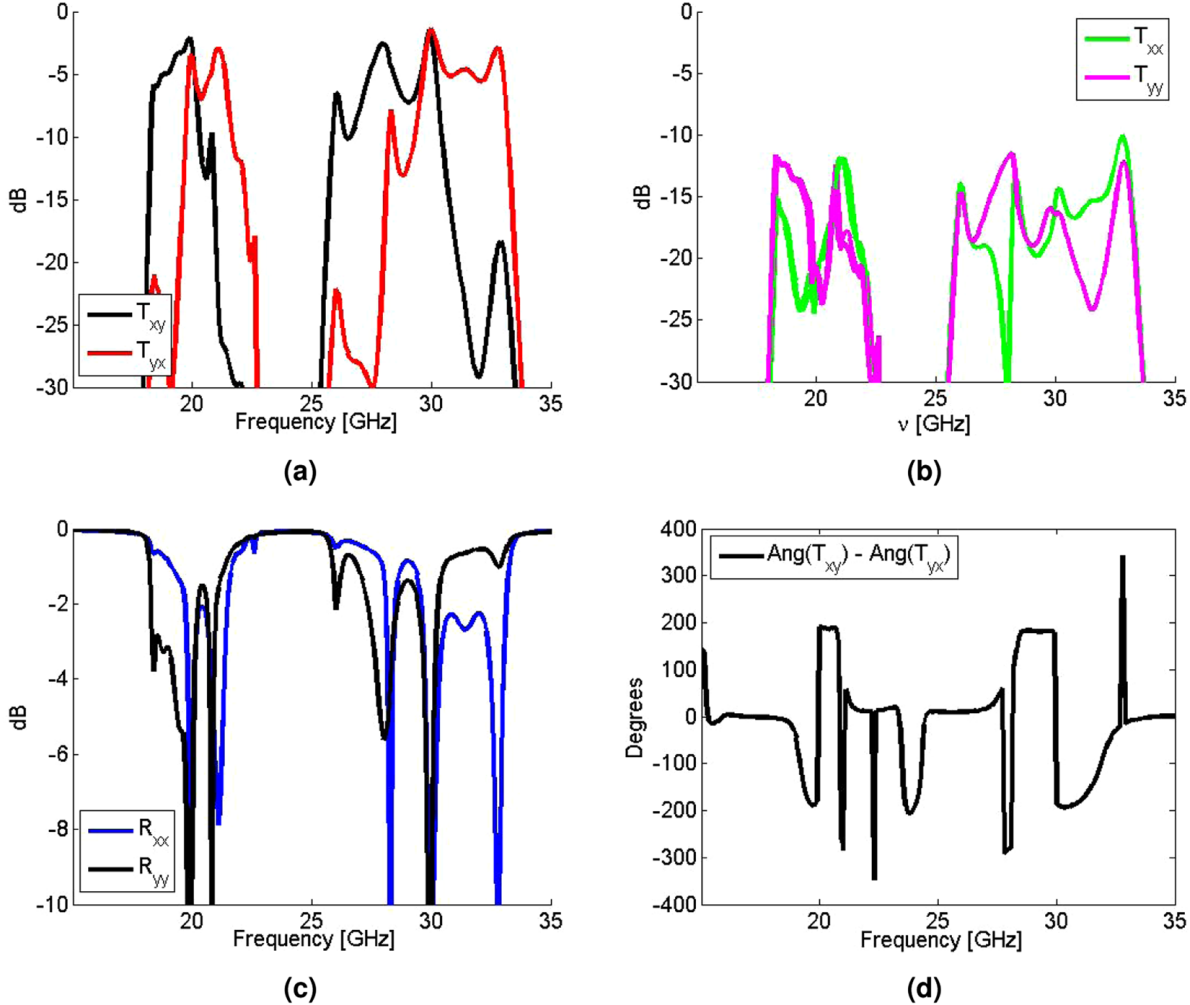
Simulation results of the dual-band 90° polarization rotator showing the (**a**) cross-polarized transmission, (**b**) co-polarized transmission, (**c**) co-polarized reflection, and (**d**) the phase difference between the two cross-polarized transmitted waves.

A similar approach has been taken to numerically verify the performance of the proposed dual-band PR. Figure 4a shows that the cross-polarized transmission of a linearly polarized wave is maximized at 20 GHz and 30 GHz, while the co-polarized transmission and reflection coefficients are well below −10 dB at these frequencies as shown in Fig. 4b and c. Specifically, the cross-polarized transmission coefficients of a *x*-polarized wave (T_*xy*_) at 20 GHz and 30 GHz are −2.11 dB and −1.72 dB respectively. For the cross-polarized transmission coefficients of a *y*-polarized wave (T_*yx*_), the maximum values at 20 GHz and 30 GHz are −3.56 dB and −1.72 dB respectively. Furthermore, Fig. 4d shows that the phase difference between the two cross-polarized transmitted fields are nearly 180° as desired (190° at 20 GHz and −185° at 30 GHz).

### Experimental Verification of the Dual-band CPSS

**Figure 5:**
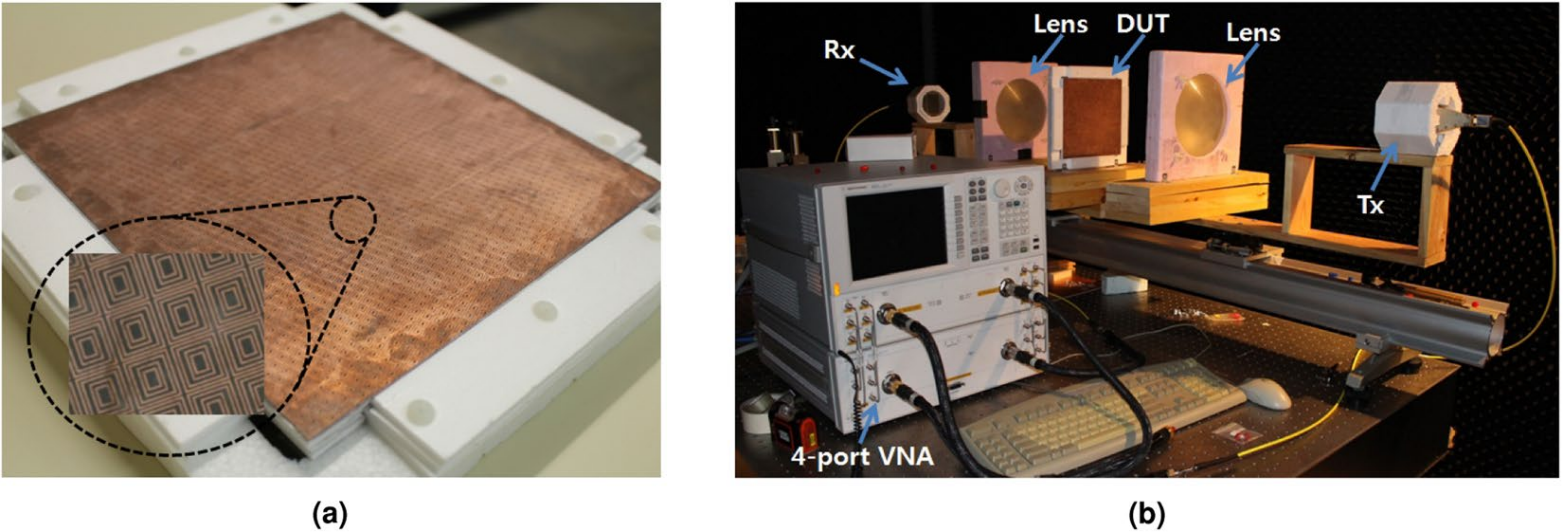
(**a**) The fabricated dual-band CPSS with the magnified view of a few arrays on the first layer and (**b**) the quasi-optical measurement setup.

To further verify the proposed dual-band chiral metasurfaces and their synthesis method, the previously designed dual-band CPSS has been fabricated and measured. The metalization has been deposited on a 0.127 mm thick Rogers 5880 substrate for all layers and each layer has been cascaded onto each other with 3-mm thick foam boards to create the necessary air gaps. Figure 5a shows the constructed dual-band CPSS. To measure its scattering properties, we have employed the free-space quasi-optical system shown in 5b. The system consists of transmitting (Tx) and receiving (Rx) horn antennas with two lenses in between. The dual-band CPSS (DUT) is placed between the two lenses as shown in the figure. The output of each horn antenna is modeled as a Gaussian beam to determine the optimal distance between the horn antennas and lenses^28^ which are computed to be 130 mm and 480 mm at 20 GHz and 30 GHz respectively. Each port of the antennas is connected to a 4-port vector network analyzer (Agilent Technologies E8361C connected to the S-parameter test set, N4421B). Hence the set up is fully vectorial and a 4 × 4 S-matrix can be measured in a single pass provided that the Tx and Rx antennas are dual-polarized. For the measurement near 20 GHz, we have actually utilized dual-polarized horn antennas. However, single-polarized horn antennas have been used at 30 GHz due to lack of dual-polarized horns. Nonetheless, by rotating either the Tx or the Rx antenna by 90°, the complete transmission properties can be measured.

**Figure 6:**
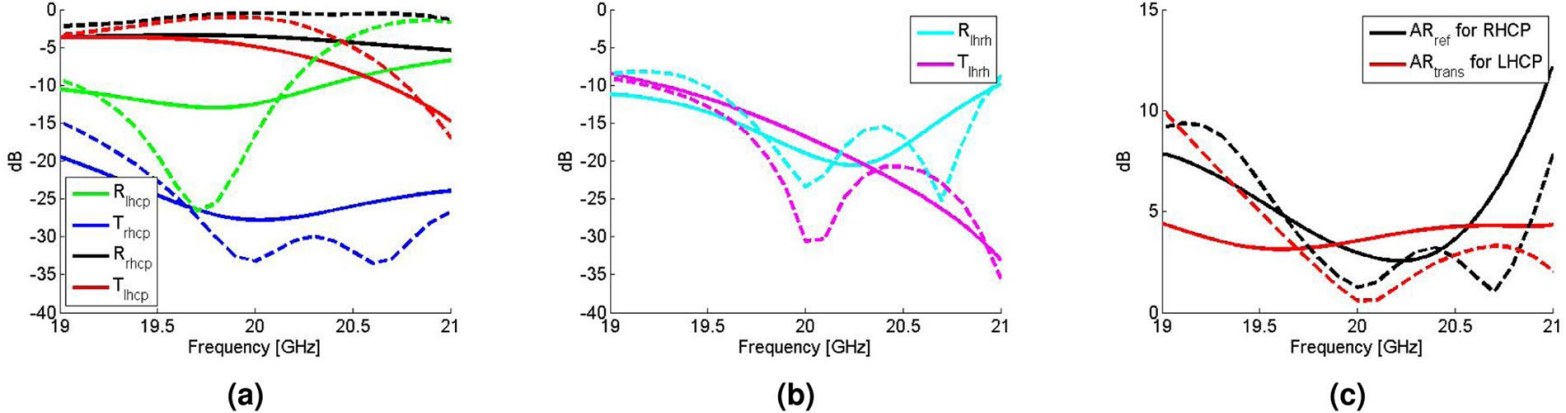
The measurement results near *f*_1_ = 20 GHz. The solid lines are measured values, whereas the dashed lines are corresponding simulated values. (**a**) The co-polarized reflection and transmission coefficients of LHCP and RHCP waves and (**b**) the cross-polarized transmission and reflection coefficients. The subscript in the cross-polarized coefficients, lhrh and rhlh, respectively refer to LHCP-to-RHCP and RHCP-to-LHCP. (**c**) The axial ratio of the reflected RHCP wave (AR _ref_ for RHCP) and the transmitted LHCP wave (AR_trans_ for LHCP).

Based on the calibrated set up (see Methods), the time-gated co-polarized and cross-polarized reflection and transmission coefficients are first measured near 20 GHz. The measured S-parameters are then converted in terms of the CP basis and Fig. 6a,b,c summarize the results. Figure 6a shows that the co-polarized transmission of LHCP (T_lhcp_) and reflection of RHCP (R_rhcp_) are maximized near 20 GHz (T_lhcp_ = −4.8 dB and R_rhcp_ = −3.5 dB at 20 GHz), while the co-polarized reflection of LHCP (R_lhcp_) and transmission of RHCP (T_rhcp_) are all below −10 dB. Furthermore, the cross-polarized reflection and transmission coefficients are also well below −10 dB near 20 GHz as shown in Fig. 6b. These results indicate that the devised surface is well-matched to its terminating medium. Figure 6c further confirms that the measured axial ratio of the transmitted LHCP and reflected RHCP are in general agreement between the simulated values, and are 3.55 dB and 2.95 dB respectively at 20 GHz.

**Figure 7:**
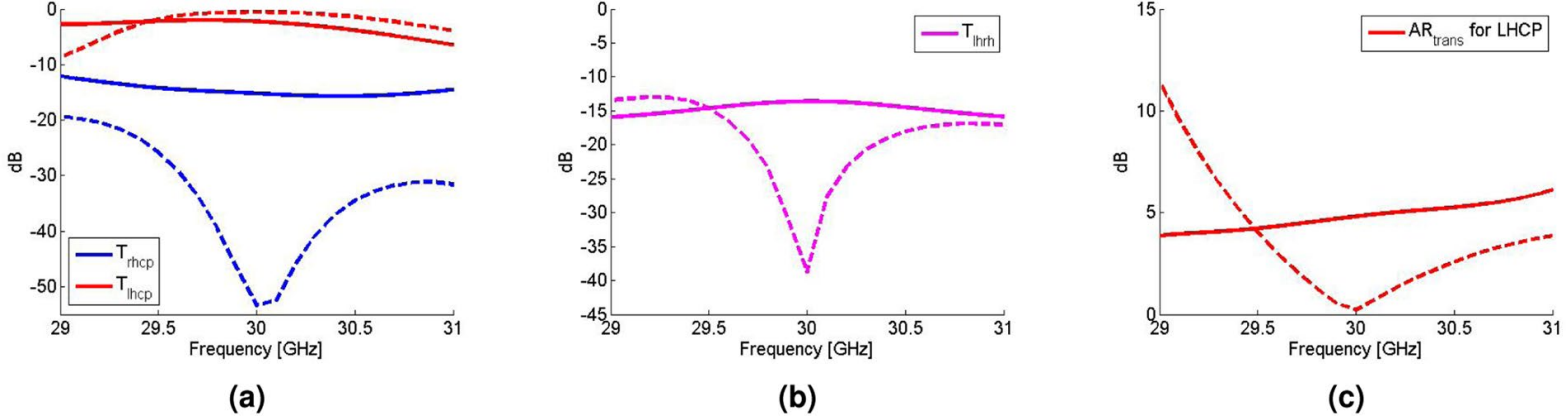
The measurement result near *f*_2_ = 30 GHz. The solid lines are measured values, whereas the dashed lines are corresponding simulated values. (**a**) The co-polarized transmission coefficients of LHCP and RHCP waves and (**b**) the cross-polarized transmission coefficients. The subscript in the cross-polarized coefficients, lhrh, represents LHCP-to-RHCP. (**c**) The axial ratio of the transmitted LHCP wave (AR_trans_ for LHCP).

The same quasi-optical set up has been used to measure the dual-band CPSS near 30 GHz, but with single-polarized horn antennas. Since the horn antennas are single-polarized, it is not feasible to measure the cross-polarized reflection coefficients. Nonetheless, full transmission properties can still be obtained from two measurements in which the Tx and Rx antennas are aligned in the first measurement whereas the Rx antenna is rotated by 90° in the second measurement. Figure 7a and b respectively show the co-polarized and cross-polarized transmission coefficients in the CP basis, while Fig. 7c shows the axial ratio of the transmitted LHCP wave. Again, the co-polarized transmission of LHCP is maximized near 30 GHz (T_lhcp_ = −2.2 dB at 30 GHz), while the other transmission coefficients are minimized and are all below −10 dB. However, the measured axial ratio at 30 GHz (4.8 dB) deviates further from the corresponding simulated value (0.21 dB) compared to the deviation at 20 GHz. In what follows, the discrepancy between the measured and simulated values is discussed in detail.

The measurements at 20 GHz and 30 GHz show that the fabricated surface indeed functions as an impedance-matched dual-band CPSS. However, it is not perfect as there are some discrepancies between the measured and simulated values. In particular, at 20 GHz, the measured transmission of the LHCP wave is −4.8 dB, whereas the corresponding simulated value is −1.1 dB. Furthermore, the simulated minimum of the cross-polarized transmission coefficient at 20 GHz is not observed in the measurements despite the fact that the measured value is still small (−16.8 dB or 0.145 in the linear scale). On the other hand, at 30 GHz, the measured axial ratio of the transmitted LHCP wave is 4.6 dB, while the corresponding simulated value is 0.21 dB. Moreover, the measured cross-polarized transmission coefficient is maximized at 30 GHz in the measurement window (29 GHz–31 GHz) although the corresponding simulated value is minimized at 30 GHz. The Supplemental Information analyzes two main sources of these discrepancies: fabrication imperfections and Ohmic losses. The fabrication imperfections include a physical variation in the actual unit cell geometries and an increase in the separation length between each layer due to the finite thickness of the double-sided tapes used between layers. These variations alter the spectral responses rather significantly and affect the overall performance of the device (see Supplemental Information). Moreover, the metallic Ohmic loss pertaining to the device sensitivity also affects the overall device performance. In particular, it predominantly affects the discrepancies in the co-polarized transmission of the LHCP waves. This is also seen from Figs 6 and 7 because the cross-polarized transmission and reflection between LHCP and RHCP and the co-polarized reflection of the RHCP waves are all small (below −10 dB). It should be noted that the discrepancy in the co-polarized transmission coefficient of the LHCP wave is greater at 20 GHz compared to the discrepancy at 30 GHz. This is because the unit cells are operating closer to their first resonance at 20 GHz and they are electromagnetically smaller compared to the ones at 30 GHz. Therefore, any small physical deviation in the fabricated sample makes the unit cells to operate even closer to their first resonance at 20 GHz, thereby resulting to a higher overall loss and consequently higher discrepancy. Furthermore, surface roughness effectively reduces the conductivity of copper that also results to a higher overall loss in the measurements. Once the aforementioned variations are taken into account, the measured values at 20 GHz and 30 GHz approach closer to the simulated ones (see Supplemental Information). Lastly, a measurement error can also occur during the calibration process if a metal plate has been misplaced even by 1 mm when defining the reflect standard or if a quarter-wavelength line has not been consistent during the calibration process. A physical misplacement of 1 mm translates to 36° of artificial phase difference at 30 GHz. As a result, the error in the axial ratio is amplified because it is very sensitive to the relative phase between the two orthogonal linearly-polarized waves. All these factors contributed to the mismatch between the measured and simulated values and are areas of further investigation in manufacturing these surfaces.

## Discussion

We have introduced a new family of general impedance-matched dual-band chiral metasurfaces. The proposed numerical design process combines semi-analytical and nonlinear optimization methods and allows solving for the required surface impedances in each layer at two user-defined operating frequencies. These layers are cascaded together to form the desired dual-band chiral metasurface. To physically encode the required surface impedances, multiple concentric rectangular rings have been proposed as the unit cell comprising the surface. We have shown that these physical surfaces exhibit distinct dual-band resonances. Furthermore, it has been shown that these resonant frequencies can also be independently and arbitrarily tuned for two orthogonal linearly-polarized waves. To demonstrate the versatility and generality of our approach, two design examples of an impedance-matched dual-band CPSS and PR operating at 20 GHz and 30 GHz have been demonstrated numerically. The impedance-matched dual-band CPSS has been further confirmed experimentally with free-space quasi-optical measurements. Good agreement between the experimental and simulation results has been achieved.

## Methods

### Simulation of the unit cell

Full-wave electromagnetic simulations are performed using the Ansoft High Frequency Structure Simulator (HFSS) commercial software. The conductivity of 5.8 × 10^7^ Siemens/m is set for the copper. A single unit cell is simulated in a three dimensional environment by assigning Floquet ports on its top and bottom surfaces for a normally incident plane-wave excitation and terminating its sides by periodic boundary conditions to simulate an infinite array. The shunt impedances of an infinite array of the unit cells are directly related to the Z-parameters that HFSS computes as,

15 $${{\rm{Z}}}_{ij}^{{\rm{sh}}}={{\rm{Z}}}_{{\rm{HFSS}}}\mathrm{(2}i1j),$$

### Calibration of the quasi-optical measurement set up

The standard four-port and two-port TRL calibrations are respectively performed for the measurements near 20 GHz and 30 GHz. A metal plate is used as the reflect standard and the reference planes of the sample are defined as thru. The horn antennas, lenses, and the DUT are all placed on micrometer translation stages such that the free-space quarter-wavelength line can be accurately defined as the line standard. Furthermore, to filter out the unwanted reflection from the lenses and horn antennas, time gating has been applied for all S-parameter measurements^20^. We note that care must be taken when positioning the metal plate to define the reflect standard for Tx and Rx antennas especially at high frequencies. Similarly, one must ensure using a consistent line standard (the free-space quarter-wavelength line) for all ports. This is because any slight deviation translates to large artificial reflection and transmission phases which have strong influence on the measured values especially on the axial ratio measurements.

## Electronic supplementary material

Supplemental Information

## References

[1] Semchenko IV, Khakhomov SA, Samofalov AL (2010). Helices of optimal shape for nonreflecting covering. Eur. Phys. J. Appl. Phys..

[2] Yu N (2011). Light propagation with phase discontinuities: generalized laws of reflection and refraction. Science.

[3] Selvanayagam M, Eleftheriades GV (2013). Discontinuous electromagnetic fields using orthogonal electric and magnetic currents for wavefront manipulation. Opt. Express.

[4] Yu N (2013). Flat optics: Controlling wavefronts with optical antenna metasurfaces. IEEE Journal of Selected Topics in Quantum Electronics.

[5] Monticone F, Estakhri NM, Alù A (2013). Full control of nanoscale optical transmission with a composite metascreen. Phys. Rev. Lett..

[6] Kim M, Wong AMH, Eleftheriades GV (2014). Optical Huygens’ metasurfaces with independent control of the magnitude and phase of the local reflection coefficients. Phys. Rev. X.

[7] Lin D, Fan P, Hasman E, Brongersma ML (2014). Dielectric gradient metasurface optical elements. Science.

[8] Wong JPS, Epstein A, Eleftheriades GV (2016). Reflectionless wide-angle refracting metasurfaces. IEEE Antennas and Wireless Propagation Letters.

[9] Yu, N. *et al*. A broadband, background-free quarter-wave plate based on plasmonic metasurfaces. *Nano Letters* **12**, 6328–6333 PMID: 23130979 (2012). 10.1021/nl303445u 23130979

[10] Pors A, Bozhevolnyi SI (2013). Efficient and broadband quarter-wave plates by gap-plasmon resonators. Opt. Express.

[11] Wu X (2016). Anisotropic metasurface with near-unity circular polarization conversion. Applied Physics Letters.

[12] Sanz-Fernández J, Saenz E, de Maagt P (2015). A circular polarization selective surface for space applications. IEEE Transactions on Antennas and Propagation.

[13] Mohamad S, Momeni A, Abadi H, Behdad N (2016). A broadband, circular-polarization selective surface. Journal of Applied Physics.

[14] Zhao Y, Belkin MA, Alù A (2012). Twisted optical metamaterials for planarized ultrathin broadband circular polarizers. Nat Commun.

[15] Wang Y-H (2016). Unidirectional cross polarization rotator with enhanced broadband transparency by cascading twisted nanobars. Journal of Optics.

[16] Gansel JK (2009). Gold helix photonic metamaterial as broadband circular polarizer. Science.

[17] Ericsson, A. & Sjoberg, D. Design and analysis of a multilayer meander line circular polarization selective structure. *IEEE Transactions on Antennas and Propagation* (In print), 10.1109/TAP.2017.2710207.

[18] Masud, M. M., Ijaz, B., Iftikhar, A., Rafiq, M. N. & Braaten, B. D. A reconfigurable dual-band metasurface for EMI shielding of specific electromagnetic wave components. In *2013 IEEE International Symposium on Electromagnetic Compatibility* 640–644, 10.1109/ISEMC.2013.6670490 (2013).

[19] Lundgren, J. *Dual Band Circular Polarization Selective Structures for Space Applications*. Master’s thesis, Lund University (2016).

[20] Selvanayagam M, Eleftheriades GV (2016). Design and measurement of tensor impedance transmitarrays for chiral polarization control. IEEE Transactions on Microwave Theory and Techniques.

[21] Kim M, Eleftheriades GV (2016). Highly efficient all-dielectric optical tensor impedance metasurfaces for chiral polarization control. Opt. Lett..

[22] Pfeiffer C, Zhang C, Ray V, Guo LJ, Grbic A (2014). High performance bianisotropic metasurfaces: Asymmetric transmission of light. Phys. Rev. Lett..

[23] Pfeiffer C, Zhang C, Ray V, Guo LJ, Grbic A (2016). Polarization rotation with ultra-thin bianisotropic metasurfaces. Optica.

[24] Kim, M. & Eleftheriades, G. V. Dual-band chiral metasurfaces. In *2017 IEEE International Symposium on Antennas and Propagation USNC/URSI National Radio Science Meeting* 1491–1492 (2017).

[25] Potter JE (1966). Matrix quadratic solutions. SIAM Journal on Applied Mathematics.

[26] Bhattacharyya, A. K. *GSM Approach for Multilayer Array Structures* 187–226 (John Wiley & Sons, Inc., 2006).

[27] Ryan CGM (2010). A wideband transmitarray using dual-resonant double square rings. IEEE Transactions on Antennas and Propagation.

[28] Goldsmith, P. F. *Gaussian Beam Coupling to Radiating Elements*, 157–185 (Wiley-IEEE Press, 1998).

