# Supplementary material for: Design and Demonstration of Impedance-matched Dual-band Chiral Metasurfaces
Source: Sci Rep. 2018 Feb 22;8:3449. doi: 10.1038/s41598-018-20056-2 (PMC5823897; doi:10.1038/s41598-018-20056-2)
Supplement: Supplementary file 1 — Supplemental Information [file 41598_2018_20056_MOESM1_ESM.pdf]

Design and Demonstration of Impedance-matched Dual-band Chiral Metasurfaces

Supplemental Information

Minseok Kim, George V. Eleftheriades[1*]

1The Rogers S. Sr. Department of Electrical and Computer Engineering, University of Toronto, King's College Rd., Toronto, ON, M5S 3G4, Canada.

Minseok Kim (minseok.kim@mail.utoronto.ca)

George V. Eleftheriades (gelefth@waves.utoronto.ca)

Effects of Physical Perturbation on Device Performance

In this Supplemental Information, we explore the effects of (a) fabrication imperfections and (b) Ohmic losses on the performance of the devised dual-band CPSS. We note that, by fabrication imperfections, we specifically mean the variation in the actual unit-cell geometries and an increase in the separation length between each layer. To take these variations into account, each layer is numerically simulated again with reduced copper conductivity (10 times lower than that of the bulk conductivity) to model the surface roughness. Furthermore, the values of ideal surface impedances and the separation lengths between each layer are increased by 15% to mimic fabrication imperfections and to incorporate the thickness of double-sided tapes used between layers. Figures S1 and S2 show the results of these variations near 20 GHz and 30 GHz.

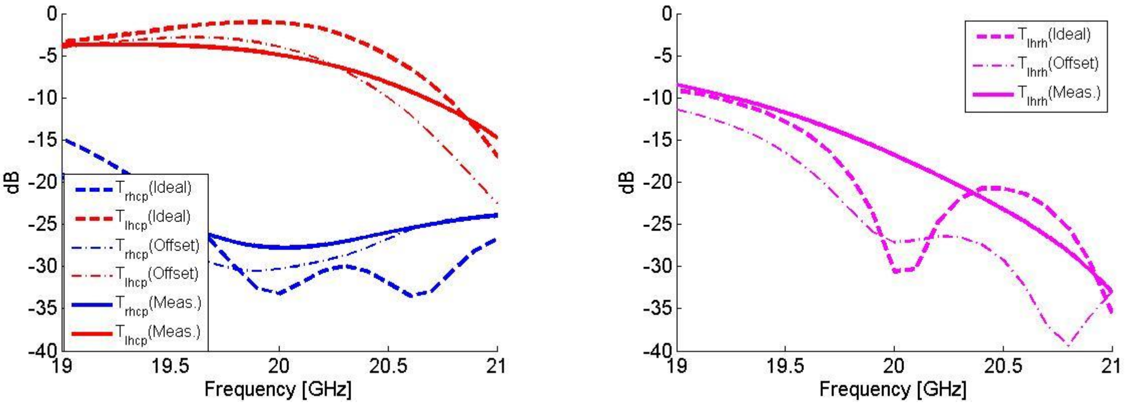

(a) Plot of T_{lhcp} (red) and T_{rhcp} (blue) **(b) Plot of T_{lhrh}**

Figure S1. The effects of fabrication imperfections and Ohmic loss on the device performance near 20 GHz.

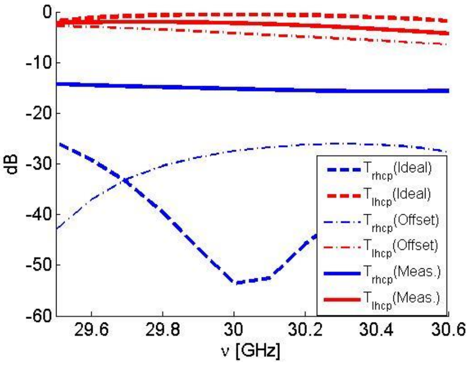

(a) Plot of T_{lhcp} (red) and T_{rhcp} (blue)

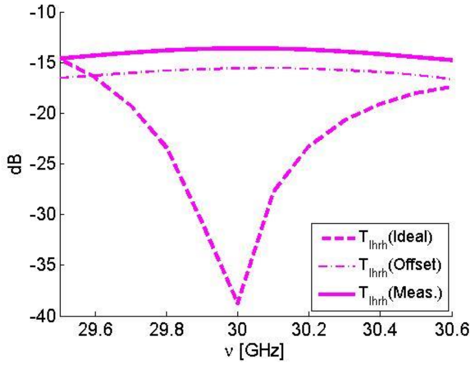

(b) Plot of T_{lhrh}

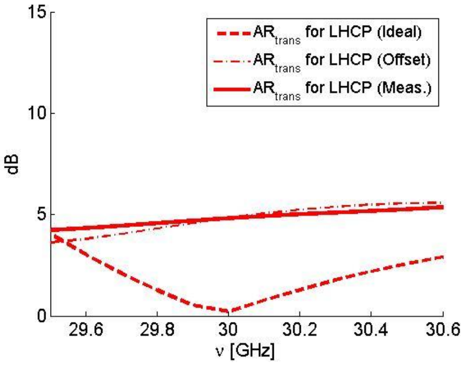

(c) Plot of AR_{trans} for LHCP

Figure S2. The effects of fabrication imperfections and Ohmic loss on the device performance near 30 GHz.

In Figures 1S and 2S, the solid lines are the measured values, whereas the thick dashed lines represent the ideal semi-numerical results that have been presented in the original manuscript (i.e., each ideal layer is numerically simulated and analytically cascaded to compute the overall S-matrix). The thin dash-dot lines represent the second semi-numerical result that takes into account the aforementioned variations. From these figures, it is seen that, although variations can be small (a 15% increase from the ideal values), they have a rather significant impact on the overall performance. Specifically, the difference between the ideal and measured co-polarized transmission coefficients of the LHCP wave at 20 GHz is 3.7 dB, whereas the difference between the offset and measured result is reduced to 0.97 dB. In addition, the strong resonance shown in the cross-polarized transmission coefficients near 20 GHz (i.e., the dip shown in T_{lhrh} in the ideal case) starts to vanish as the variations are taken into account and approaches closer to that of measured values. The difference between the measured and simulated values becomes smaller if higher variations are applied.

The difference between the measured and simulated values becomes even closer at 30 GHz.

In particular, it has been found that the variation in the separation lengths between each layer has more predominant effects than the variation in surface impedances at 30 GHz. This is understandable considering the frequency of operation. Although an increase of 15% in the separation lengths translates to merely a 0.45 mm increase, the change in its electrical length at 30 GHz corresponds to 16° of phase alteration in *each* layer. Since the waves bounce back and forth between the layers, such a phase discrepancy accumulates and gets amplified, thereby affecting the overall performance. Indeed, the simulated results for the offset design matches very well the measured values (e.g., AR_{trans} for LHCP and T_{lhrh}).
